# Benefits of Wise Organizations on Employees’ Well-Being: A Comparison of Younger and Older Workers

**DOI:** 10.1093/geroni/igab046.3283

**Published:** 2021-12-17

**Authors:** Monika Ardelt, Bhavna Sharma

**Affiliations:** University of Florida, Gainesville, Florida, United States

## Abstract

Research has shown a positive relation between personal wisdom and well-being, particularly in old age. Yet, it is unknown whether wisdom in the workplace also has a positive impact on workers’ well-being. We created a wise organization index for nine organizations based on 74 to 390 average employees’ ratings of perceived flexibility at work, work opportunities for training and development, satisfaction with work benefits, absence of time pressure at work, work-life balance, job fulfillment, and job security. We predicted a stronger relation of wise organization on well-being for older workers (N=269; age range 50-74, M=56.08, SD=5.04) than for younger workers (N=552; age range 19-49, M=35.10, SD=8.17) who can more easily change jobs. Results of multigroup analyses in LISREL 9.30 showed that the wise organization index had significant indirect effects on employees’ physical and subjective well-being at the second wave of data collection, mediated by employees’ perception of wise (fair and supportive) leadership assessed six months earlier and overall work satisfaction (career as calling, satisfaction with career progress, enthusiasm at work, and great workplace) at Wave 2. Contrary to expectations, the effects were not statistically different between the two age groups. However, physical well-being had a statistically stronger association with subjective well-being among younger rather than older workers, possibly indicating a positive health selection effect in the older age group. It appears that wise organizations encourage wise leadership and enable workers to work longer by contributing to their work-related, physical, and subjective well-being.

